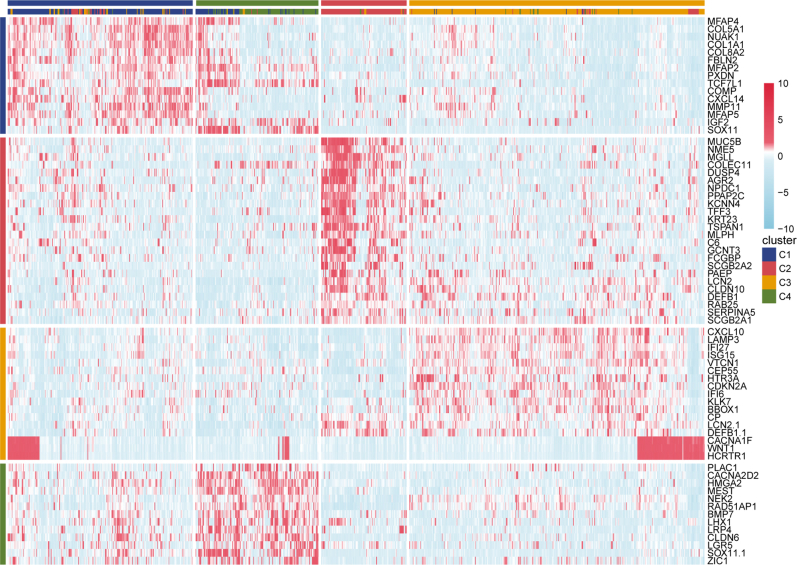# Comprehensive analysis of consensus molecular subtypes for ovarian cancer from bulk to single-cell perspectives

**DOI:** 10.1016/j.jbc.2024.107710

**Published:** 2024-08-22

**Authors:** Ziyan Zhao, Linan Xing, Qian Cheng, Zhiyi Wu, Fei Xue, Yunyi Peng, Yuxi Zhang, Guixiang Lv, Yongjian Zhang, Chunlong Zhang

**Affiliations:** 1 College of Bioinformatics Science and Technology, Harbin Medical University, Harbin, China; 2 Department of Gynecology, The First Affiliated Hospital, Zhejiang University School of Medicine, Hangzhou, China; 3 Department of Biochemistry and Molecular Biology, Harbin Medical University, Harbin, China; 4 Department of Gynecology Oncology, Harbin Medical University Cancer Hospital, Harbin, China

**Keywords:** ovarian cancer, molecular subtypes, integrated analysis, single-cell RNA-seq

## Abstract

Molecular subtypes play a pivotal role in guiding preclinical and clinical risk assessment and treatment strategies in cancer. In this study, we extracted whole-tissue transcriptomic data from 1987 ovarian cancer patients spanning 26 independent Gene Expression Omnibus cohorts. A total of four consensus subtypes (C1–C4) were identified, notably, subtype C1 samples exhibited a poor prognosis and higher M2 macrophages infiltration, whereas subtype C2 samples demonstrated the best prognosis and higher CD4 resting T cells infiltration. Additionally, we characterized cancer- and stromal-specific gene expression profiles, and conducted an analysis of ligand–receptor interactions within these compartments. Based on cancer compartment, subtype-specific interactions as well as gene signatures for each molecular subtype were identified. Leveraging single-cell transcriptomic data, we delineated malignant epithelial cells with four molecular subtypes and observed an increase in C1 cell proportions from primary to relapse to metastasis stages, with a corresponding decrease in C2 cell proportions. Furthermore, we investigated subtype-specific interaction with T cells through integrated analysis of bulk and single-cell datasets. Finally, we developed a robust ten-gene risk model based on subtype gene signatures for prognostic evaluation in ovarian cancer, demonstrating its efficacy across independent datasets. In summary, this study systematically explored ovarian cancer molecular subtypes and provided a framework for other cancer types.

Ovarian cancer (OC) is a malignant neoplasm affecting the female reproductive system and represents a significant cause of mortality among women worldwide (1). Recent improvements in cancer survival rates can be largely attributed to pioneering research and continuous advancements in screening, surgical techniques, and therapeutic approaches. While cancer classification traditional relies on histopathological and clinical criteria, molecular data offer the potential to delineate subtypes with distinct biological characteristics and prognostic implications (2, 3). However, there remains a need for integrated analysis utilizing large-scale datasets to comprehensively define OC molecular subtypes and to explore their systemic functions.

Tumors are complex entities composed of heterogeneous populations of cancer cells and infiltrating nontumor cells, representing a fundamental characteristic of malignancy. Previous studies have focused on assessing the cellular composition within individual tumor samples (4, 5). OC patients commonly exhibit high recurrence rates, poor long-term survival, and extensive intertumoral and intratumoral heterogeneity (6). Understanding the mechanisms driving tumor heterogeneous remains a formidable challenge. Exploring tumors across different evolutionary stages and investigating their molecular heterogeneity may unveil underlying biological mechanisms (7). Recent advancements in single-cell RNA-seq have provided unprecedented insights into the transcriptomic diversity of tumors and their immune microenvironment (8, 9). Thus, a comprehensive study is needed to define the OC molecular subtypes and characterize distinct immune mechanism involved by integrating both bulk and single-cell RNA transcriptome datasets. Moreover, the large cohort study integration can enhance the rigor and reproducibility of biomarker discovery for diagnosis or prognosis analysis (10).

In this study, we integrated large-scale OC bulk transcriptome datasets to delineate consensus molecular subtypes and investigated their biological functions and network module characteristics. Using a deconvolution-based strategy on the integrated dataset, we inferred the expression profiles of cancer and stromal components across four distinct molecular subtypes. Additionally, we identified specific ligand–receptor (LR) interactions associated with each subtype. Leveraging single-cell transcriptome datasets, we characterized the malignant epithelial cells into the four identified subtypes and examined the immune associations, including interactions with immune cells such as T cells. Finally, we developed a prognostic model based on subtype specific genes, demonstrating predictive validity in the The Cancer Genome Atlas (TCGA) validation cohort. In summary, this study presents a framework for identifying and investigating OC molecular subtypes, along with the development of a novel risk model for patient prognosis and potential treatment strategies.

## Results and discussion

### Molecular subtypes identification based on bulk transcriptome datasets

**Figure 1:**
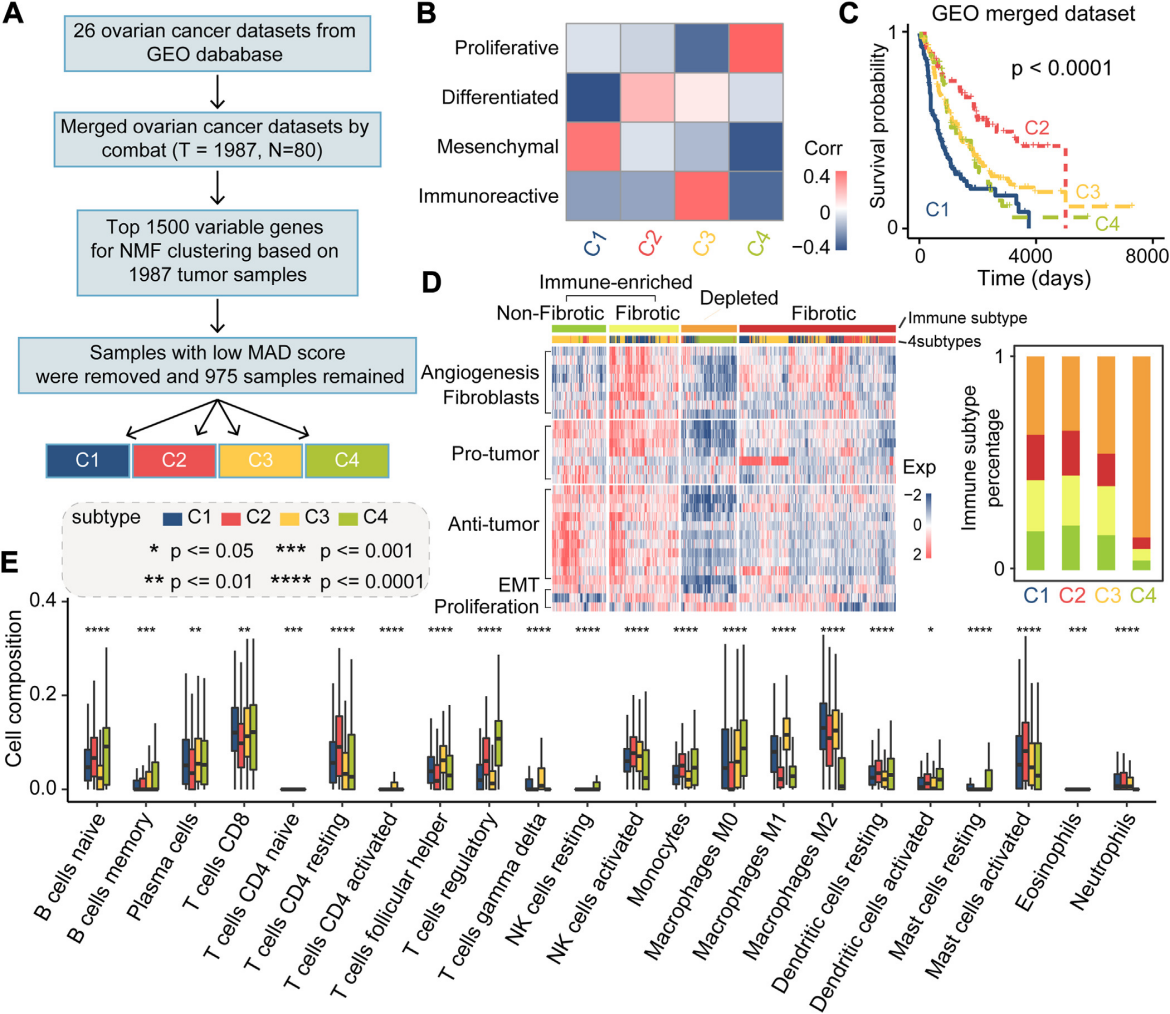
**Molecular subtypes determination and distribution of immune cell immunotypes.***A*, flowchart illustrating the process of subtype determination. *B*, correlation analysis between derived subtypes and TCGA classifications. *C*, survival analysis of four molecular subtypes. The *p* value was calculated using Log-rank test. *D*, classification of immunotypes based on subtype data and distribution of immune cell types across subtypes. *E*, distribution of various immune cell types across different subtypes. TCGA, The Cancer Genome Atlas.

**Figure S1:**
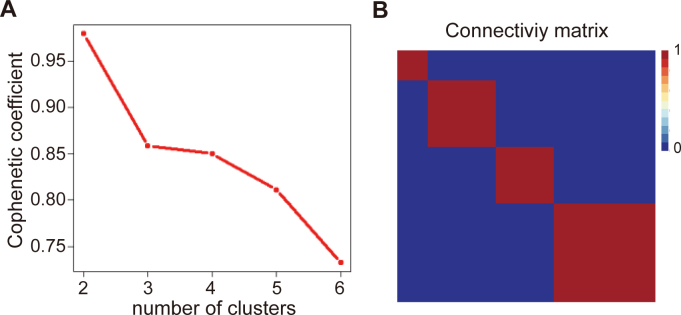


In this study, we developed a computational approach to comprehensively characterize the transcriptome and define consensus molecular subtypes by analyzing a total of 1987 OC samples sourced from 26 independent datasets (Fig. 1*A*, and Table S1). Utilizing non-negative matrix factorization clustering on the merged Gene Expression Omnibus (GEO) dataset, we constructed consensus matrices and sample correlation matrices across k-values (k = 2 to k = 6), with clustering at k = 4 demonstrating the most robust outcome (Fig. S1, Experimental procedures). Subsequently, samples with low representativeness were excluded based on silhouette width, resulting in 975 samples categorized into four consensus molecular subtypes (C1: 264, C2: 172, C3: 408, C4: 131). Furthermore, differentially expressed genes were identified within each subtype compared to the others using significance analysis of microarrays (11). F-score were computed for each gene after significance analysis of microarrays analysis and compared with TCGA subtype results, demonstrating consistent relationships between the GEO merged dataset and TCGA dataset (Fig. 1*B*). Specifically, C1 subtype was associated with a mesenchymal phenotype, C2 subtype with a differentiated phenotype, C3 subtype with an immunoreactive phenotype, and C4 subtype with a proliferative phenotype.

Furthermore, we investigated the clinical relevance of these consensus subtypes in OC survival outcomes. As shown in Figure 1*C*, samples classified under the C2 subtype exhibited the most favorable survival, whereas those in the C1 subtype displayed the poorest survival outcomes, aligning with previous observations linking C2 to a differentiated state and C1 to a mesenchymal state. Recently, researchers defined four tumor microenvironment subtypes across various cancers, including OC (12). As shown in Figure 1*D*, C4 samples were closely associated with a depleted subtype characterized by low angiogenesis and fibroblasts function, yet high activity in epithelial-to-mesenchymal transition (EMT) and proliferation. Conversely, C3 samples showed associations with an immune-enriched subtype encompassing both nonfibrotic and fibrotic types.

Based on the merged GEO dataset, we further explored the composition of immune cell infiltration in tumor samples across different molecular subtypes using the CIBERSORT method (4). Significant differences in immune cell composition were observed among the four molecular subtypes, including variations in CD4 resting T cells, regulatory T cells, M0 macrophage, M1 macrophage, and M2 macrophage (Fig. 1*E*). Notably, the activity of M2 macrophage observed in C1 samples is known to promote tumor cell proliferation (13), potentially contributing to the poorer survival outcomes observed in this subtype.

### Subtype-specific module analysis

**Figure 2:**
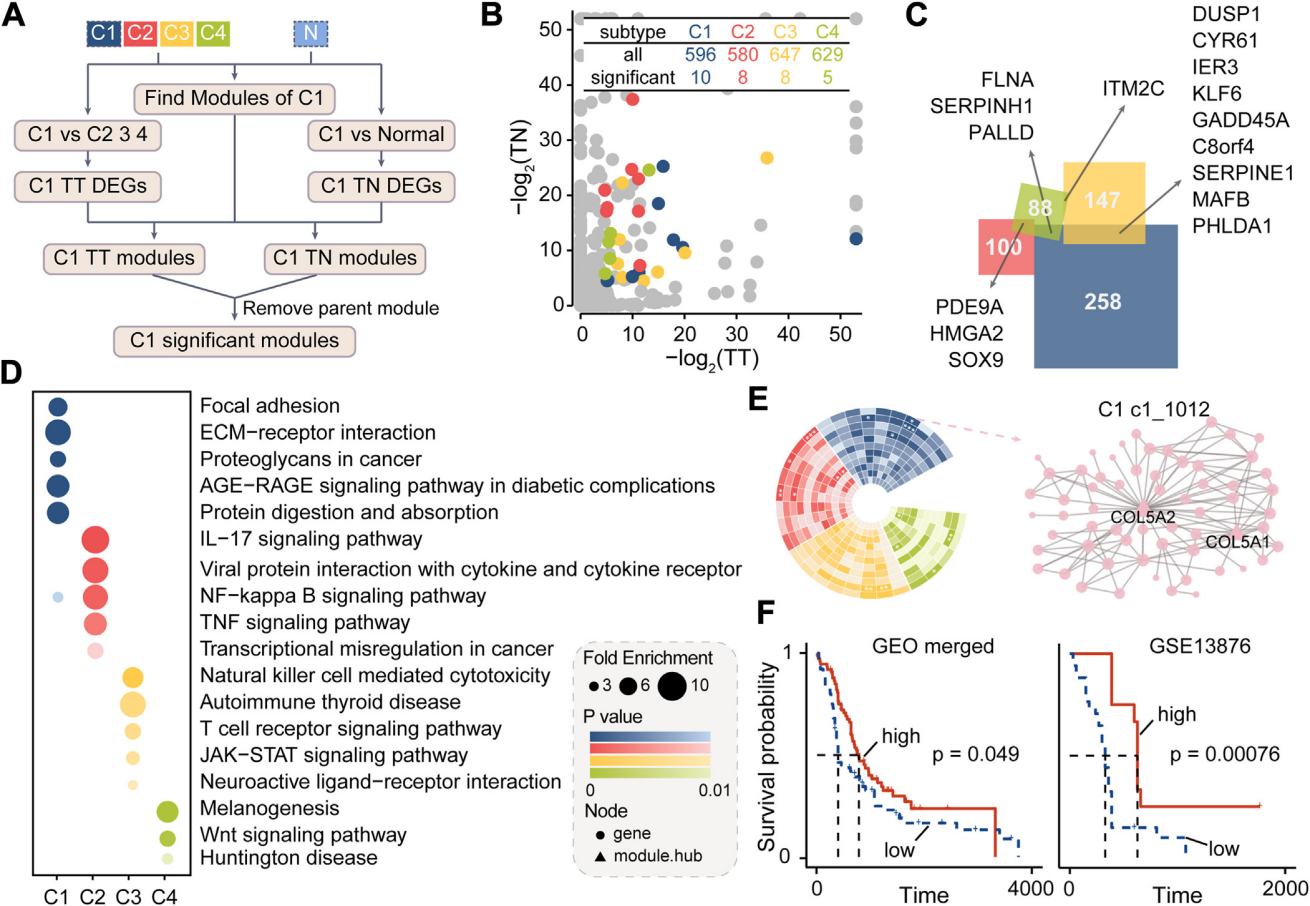
**Identification of significant modules using MEGENA.***A*, flowchart detailing the identification of significant modules (using C1 as an example, with similar procedures for other subtypes). *B*, significant modules identified for each subtype, categorized by TT *versus* TN *p* value; *gray* indicates nonsignificant modules. The table shows total module counts and significant module counts per subtype. *C* and *D*, total gene counts and top five functional enrichments of significant modules for each subtype. *E* and *F*, significant modules identified through GEO data integration and corresponding survival outcomes. The *p* value was calculated using Log-rank test. GEO, Gene Expression Omnibus.

**Figure S2:**
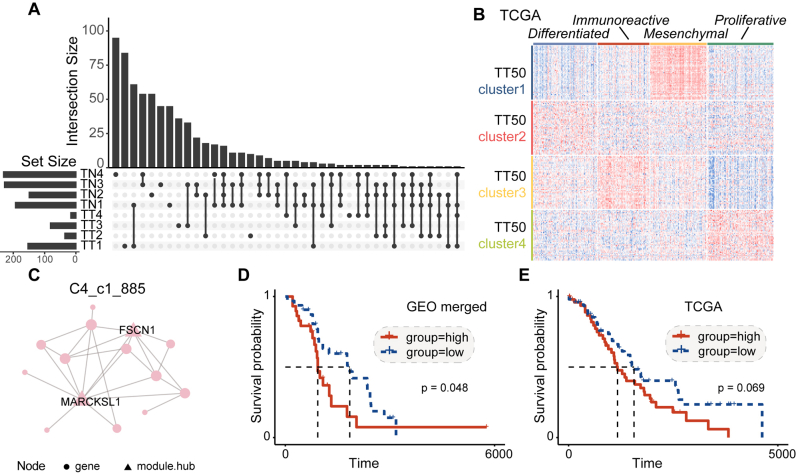


To investigate gene coexpression patterns within the four subtypes, we employed the MEGENA algorithm to identify gene modules specific to each subtype (see Experimental procedures, Fig. 2*A*). Initially, we computed two sets of differential genes: (i) genes differentially expressed (TT) between each subtype and the remaining three, (ii) genes differentially expressed (TN) between each subtype and normal samples. The associations among TT and TN genes were shown in Fig. S2*A*. Furthermore, the top 50 TT genes for each subtype consistently mirrored TCGA subtype classifications (Fig. S2*B*), underscoring the robustness of our subtype-specific gene selection.

**Figure S3:**
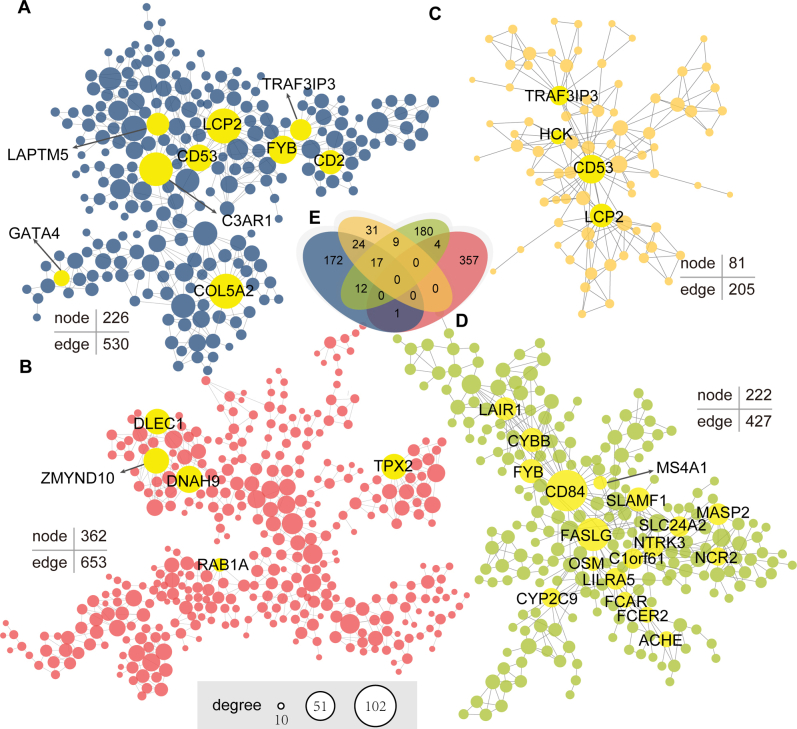


Using TT and TN subtype genes, we then delineated subtype-specific modules (ssModules), identifying 5 to 10 ssModules for C1-C4 subtypes (Fig. 2*B* and Table S2). As shown in Figure 2*C*, some genes were found across multiple ssModules, such as SOX9 and IER3. Subsequent kyoto encyclopedia of genes and genomes enrichment analysis highlighted significant pathway enrichments: the C1 module prominently featured in focal adhesion, crucial for tumor progression toward malignancy; while the C2 module showed enhanced expression in the TNF and NF-κB signaling pathways, and the roles of NF-κB signaling pathways in inflammation and cancer has been revealed (14) (Fig. 2*D*). Protein interaction networks for each molecular subtype were constructed (Fig. S3), identifying hub genes including COL5A2 (C1), LCP2 (C2), TPX2 (C3), and FASLG (C4). Furthermore, we explored the associations between these ssModules and patient survival using TCGA and GEO merged datasets (Fig. 2*E*). Several modules demonstrated prognostic relevance, such as the C1-c1-1012 module (Fig. 2*F*) and C4-c1-885 (Fig. S2, *C*–*E*). In summary, we characterized modules specific to the four molecular subtypes and identified prognostically relevant modules within C1 and C4 subtypes.

### Inference of cancer- and stromal-specific expression

**Figure 3:**
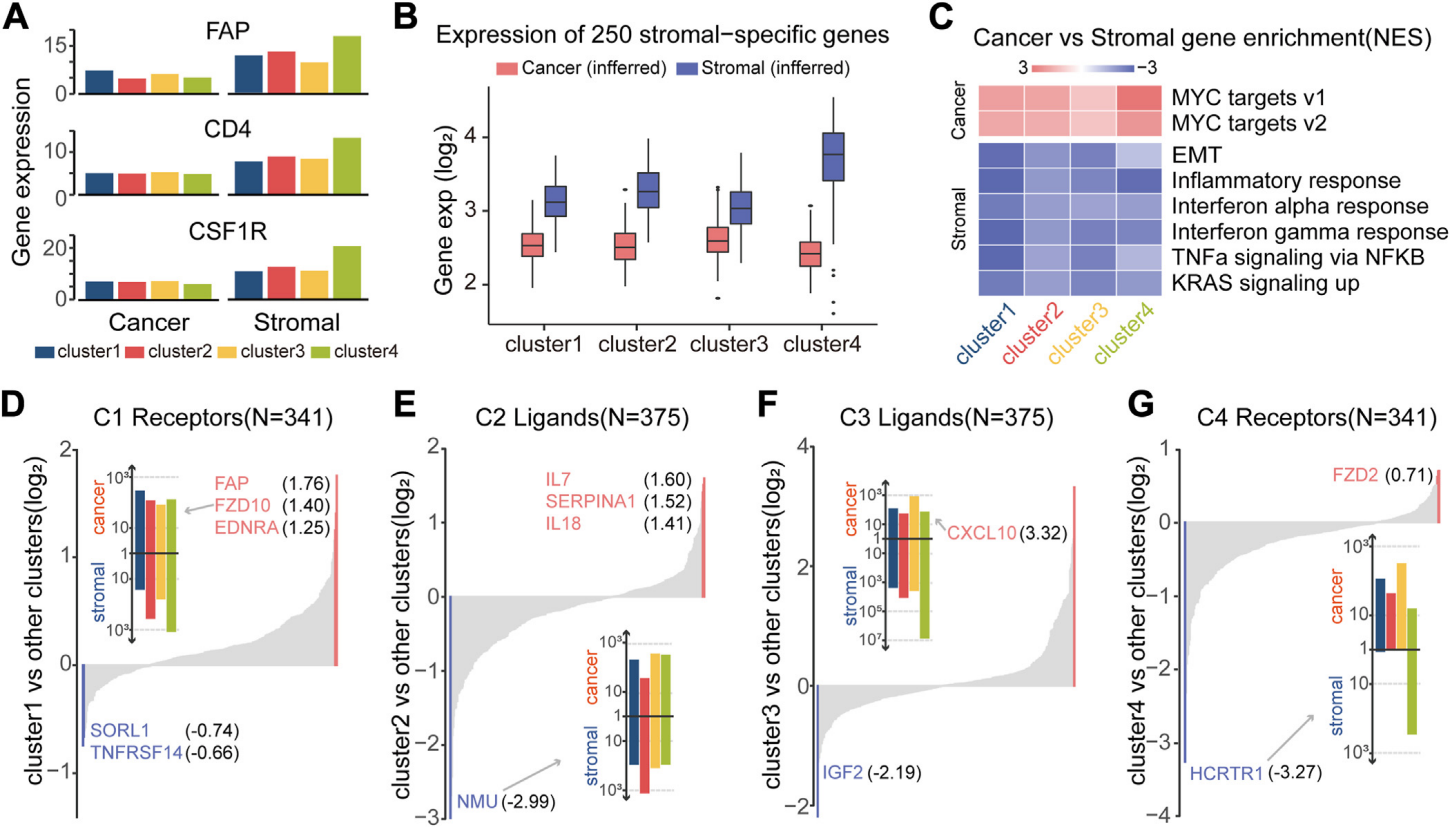
**Cancer and stromal expression profiles.***A*, expression differences of known mesenchymal genes (FAP, CD4, and CSF1R) between cancer and stromal across four subtypes. *B*, expression levels of 250 known stromal-specific genes across cancer and stromal compartments. The significance of difference between two groups was calculated using Wilcoxon rank-sum test. *C*, identified cancer and stromal-enriched gene sets using gene set enrichment analysis (GSEA) across four subtypes. *D*–*G*, expression profiles of ligands and receptors in cancer and stromal across subtypes.

In this study, we employed an algorithm to infer gene expression profiles of cancerous and stromal compartments across C1-C4 subtype samples (see Experimental procedures). As shown in Figure 3*A*, stromal markers (FAP, CD4, and CSF1R) exhibited higher expression levels in the stromal compartment than the cancerous compartment across all four subtypes. These findings were corroborated using a set of 250 stromal genes identified in previous study (15), underscoring the robustness of cancerous and stromal-specific expression patterns (Fig. 3*B*). Leveraging these specific expression profiles, we utilized gene set enrichment analysis to investigate the underlying biological mechanisms characterizing each subtype (Fig. 3*C*). Our analysis revealed significant enrichment of genes associated with cell cycle pathways in the cancerous compartments, consistent with MYC overexpression observed in OC cell lines (16). For the stromal compartments, enriched pathways included inflammatory response, interferon response, and EMT, with notable higher normalized enrichment scores observed in C1 and C3 subtypes for EMT-related pathways, consistent with previous findings (Fig. 1*D*). By integrating stromal and cancerous gene expression data, we identified common and subtype-specific gene markers across the four molecular subtypes.

**Figure S4:**
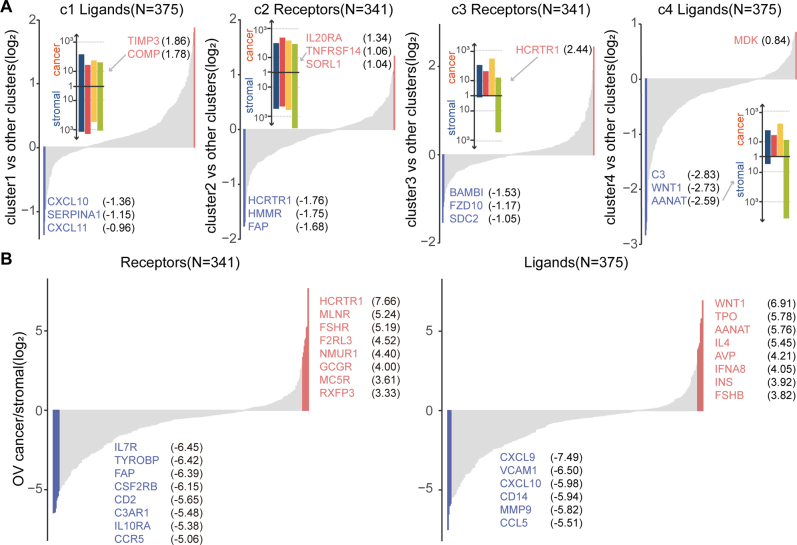


Furthermore, we computed specificity score for the ligands and receptors in both cancerous and stromal compartments (see Experimental procedures). As shown in Figures 3, *D*–*G* and S4, we identified specific ligands or receptor marker unique to each molecular subtype. For example, FZD10 showed significantly higher expression in cancer cells of C1 than other subtypes (Fig. 3*D*); whereas HCRTR1 exhibited downregulation in C2 and C4 receptors but high expression in C3 receptors (Fig. S4*A*). This suggests HCRTR1 may serve as a novel marker for distinguishing OC subtypes. Expanding our analysis, we observed specificity in 341 receptors and 375 ligands (Fig. S4*B*).

### LR interaction and immune checkpoint characterization

**Figure 4:**
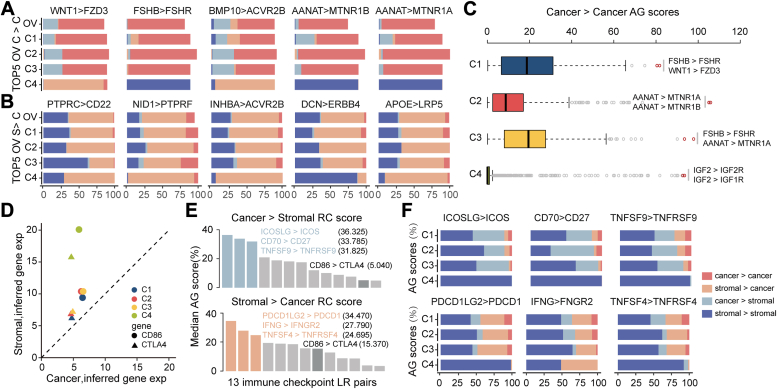
**Expression of different ligand-receptor pairs.***A* and *B*, relative crosstalk (RC score) of cancer–cancer and stromal-cancer LR pairs, focusing on the top five scoring pairs. *C*, distribution of RC scores for cancer–cancer LR pairs across subtypes, highlighting top LR pairs per subtype. *D*, expression level of immune checkpoint ligands (CTLA-4) and tis receptor (CD86) across subtypes. *E*, RC scores of cancer-stromal and stromal-cancer LR pairs, particularly highlighting known immune checkpoint pairs like CD86-CTLA4. *F*, RC scores for LR pairs with highest median scores in cancer-stromal and stromal-cancer signaling.

Based on compartment-specific expression profiles of ligands and receptors, we conducted further analysis to calculate LR relative crosstalk (RC) scores between cancer and stromal communications (see Experimental procedures). As shown in Figure 4*A*, notable receptors in cancer-to-cancer LR pairs mainly include FSHR, MTNR1B, and MTNR1A. Among these receptors, FZD3 exhibited the lowest representation in C4; decreased FZD3 expression predicts survival in cancer patients’ postchemotherapy (17). ACVR2B emerges significantly in both cancer-to-cancer and stroma-to-cancer interactions, suggesting a shared role in the transforming growth factor-β signaling pathway across the four molecular subtypes. To explore subtype-specific LR interactions, we screened the four cancer-to-cancer subtypes by LR pairs scores, as shown in Figure 4, *B* and *C*.

Immune checkpoint markers are crucial for cancer examination and treatment. Therefore, we investigated CD86>CTLA4 as a common anticancer therapeutic target to observe expression patterns across the four subtypes. Figure 4*D* shows higher stromal expression of the receptor (CTLA4) than cancer, while the ligand (CD86) demonstrated an overexpression trend, particularly in the C4 subtype. Through comprehensive score calculations, we further explored immune checkpoint characterization within the subtypes. Three LR pairs (ICOSLG>ICOS, CD70>CD27, TNFSF9>TNFRSF9) displayed high scores in cancer-to-stromal (Fig. 4*E*). ICOSLG>ICOS, an activating T cell checkpoint, exhibited heightened interaction across all subtypes except C4. Conversely, PDCD1LG2>PDCD1 emerges as most significant across several subtypes, while IFNG>IFNGR2 scored between 30% and 50% across all four subtypes (Fig. 4*F*).

### Single-cell immune microenvironment characterization

**Figure 5:**
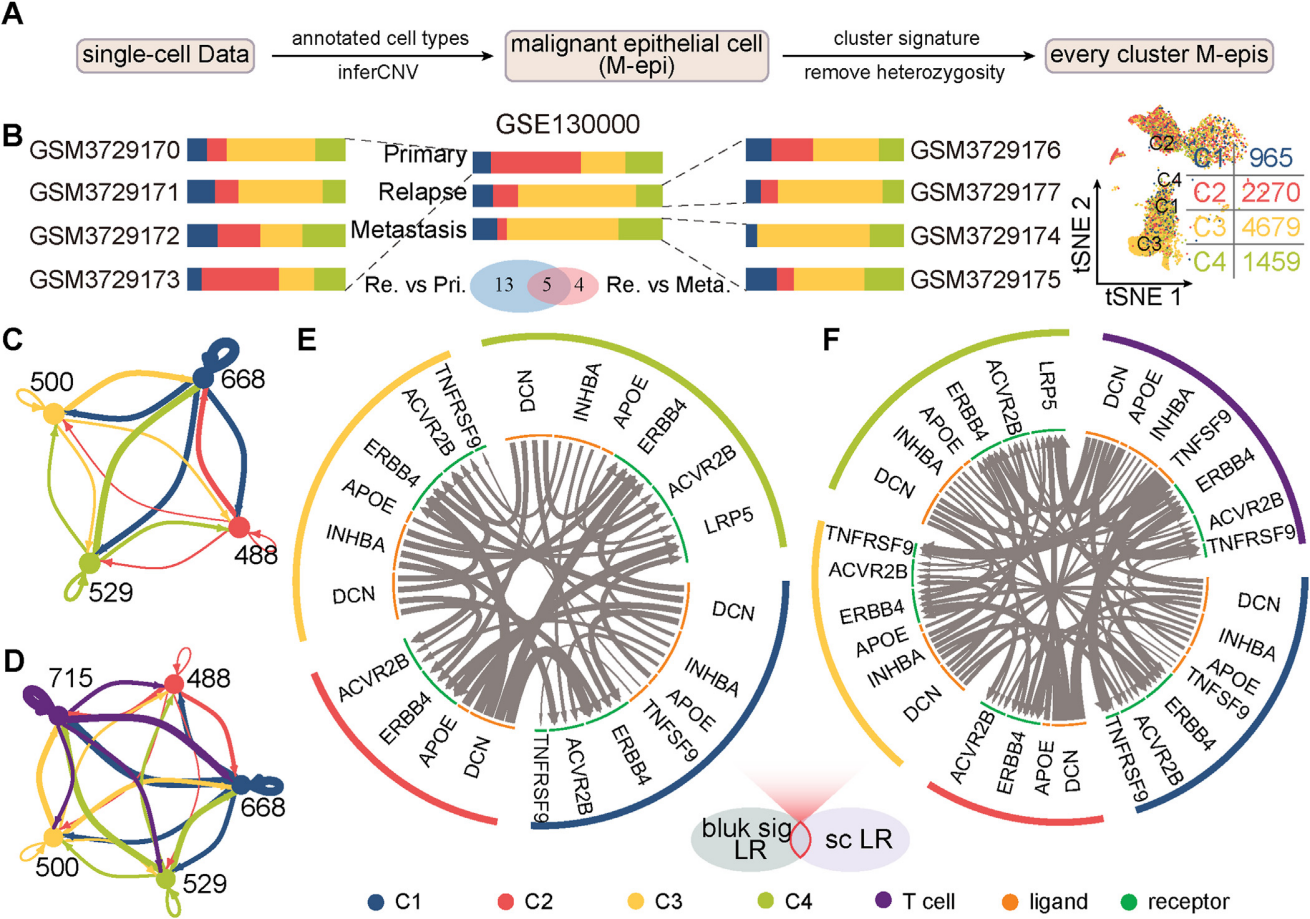
**Characterization analysis based on single-cell RNA transcriptome data.***A*, methodology for subtyping based on single-cell datasets. *B*, percentage distribution of subtypes in GSE130000 and intersection of differentially upregulated genes. *C*–*F*, interactions between subtypes and T cells, as well as ligand–receptor interactions.

**Figure S5:**
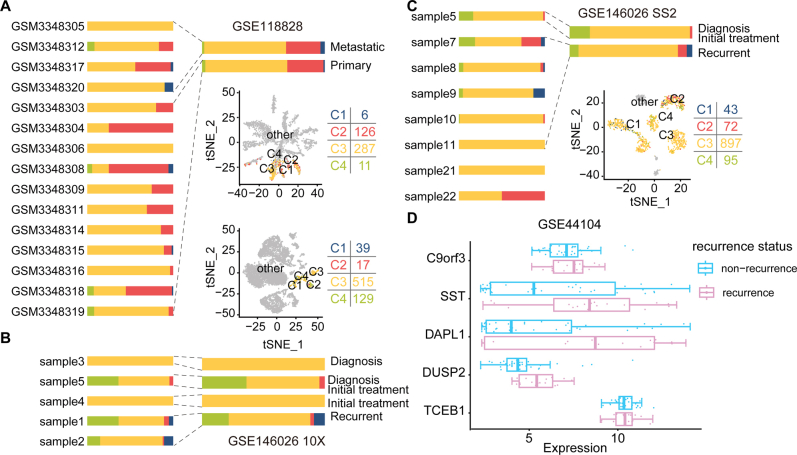


To explore interactions between malignant epithelial cells and immune cells across four molecular subtypes at the single-cell level, we curated and analyzed four OC single-cell RNA sequencing transcriptome datasets. Initially, we characterized malignant epithelial cells into different subtypes using predefined subtype signatures (see Experimental procedures, Table S3). Subsequently, each malignant epithelial cell was scored and heterogeneous cells were filtered within each subtype (see Experimental procedures, and Fig. 5*A*). Figure 5*B* illustrates a gradual increase of subtype C3 from primary to recurrence to metastasis, contrasting with a decrease of C2, potentially indicating poor prognosis for C3 and favorable prognosis for C2. Similar trends were observed across the other three single-cell RNA-seq datasets (Fig. S5, *A*–*C*).

We conducted differential expression analyses comparing relapse-primary and relapse-metastasis samples, identifying commonly upregulated genes. Notably, genes such as SST, DAPL1, and DUSP2 exhibited higher expression in recurrence samples than nonrecurrence samples (Fig. S5*D*). Furthermore, subtype-specific differential expressed genes were identified through comparisons with cells from other subtypes, with representative genes were presented in Table S4. Additionally, interactions between epithelial cells from the four subtypes and T cells were explored (Fig. 5, *C* and *D*), focusing on significant LR pairs identified from both bulk and single-cell RNA transcriptome results. Subtype-specific interactions were shown in Figure 5, *E* and *F*.

### Constructing a prognostic model based on subtype signatures

**Figure 6:**
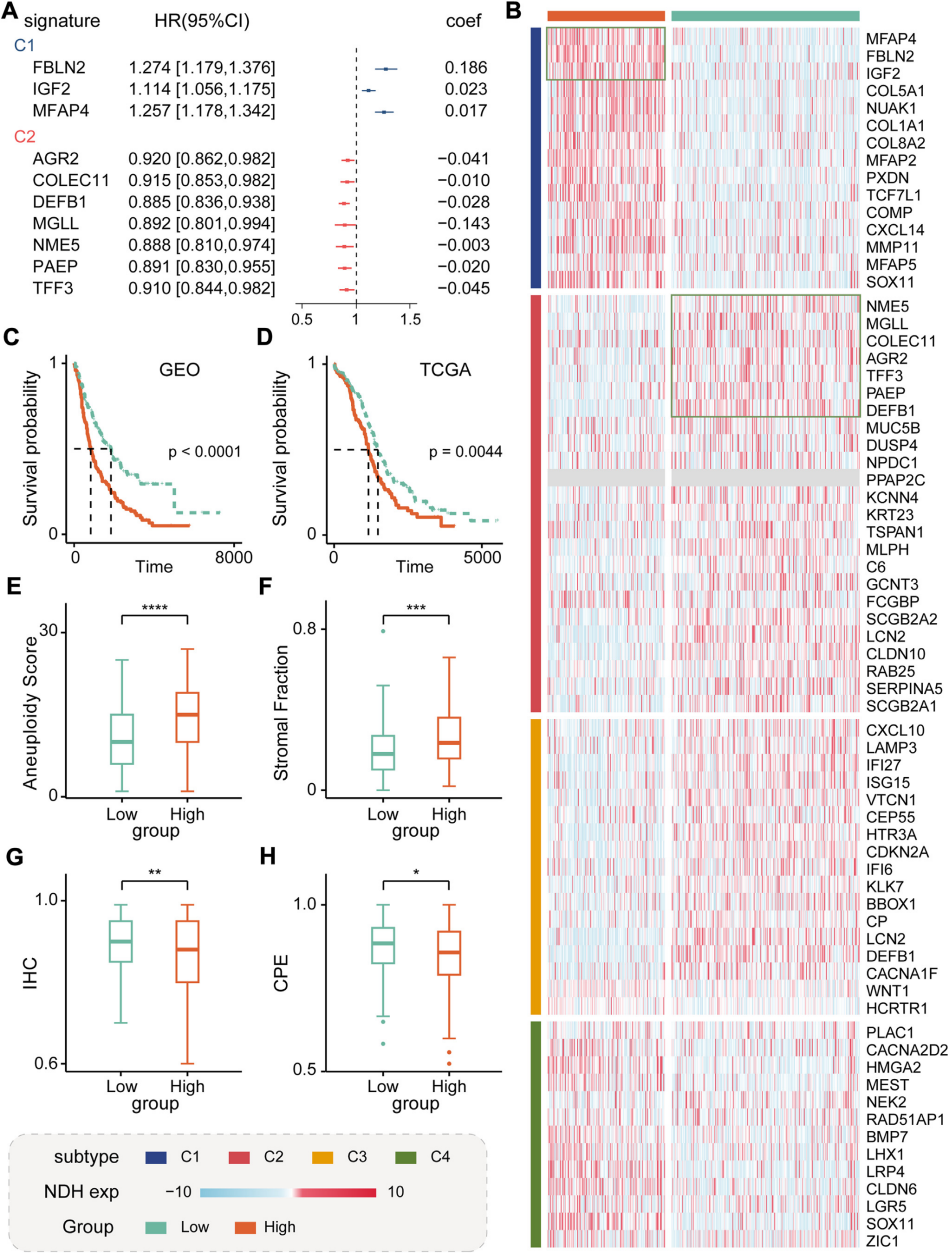
**Risk prognosis model based on subtype signatures.***A*, risk model selected based on signatures from C1 and C2 subtypes. *B*, expression profiles of all subtype signatures in TCGA, categorized by the risk score. *C* and *D*, survival analysis based on risk model in TCGA and GEO. The *p* value was calculated using Log-rank test. *E* and *F*, aneuploidy and stromal fraction expressions between high- and low-risk groups in the TCGA dataset. *G* and *H*, tumor purity comparisons using by different methods (IHC and CPE) between high- and low-risk groups in the TCGA dataset. The significance of difference between two groups was calculated using Wilcoxon rank-sum test. GEO, Gene Expression Omnibus; TCGA, The Cancer Genome Atlas. CPE, consensus measurement of purity estimations; IHC, immunohistochemistry.

Finally, we focused on two distinct subtype signatures, C1 and C2, representing the subtypes with the most favorable and the poorest survival outcomes, respectively (Figs. 1*C* and S6). Using the merged GEO dataset as our training set, we constructed risk prediction models based on signature genes from C1 and C2 using the LASSO algorithm (see Experimental procedures, Fig. 6, *A*–*C*). Subsequently, the LASSO model incorporated a total of ten genes (three from C1 and seven from C2). Genes from C1 demonstrated predictive risk performance, while those from C2 exhibited protective performance, consistent with previous observations. Based on the stratification of the training set, we partitioned the TCGA validation set into two groups, which displayed significant survival differences with a log-rank *p* value of 0.0044 (Fig. 6*D*). Concurrently, samples from the high-risk and low-risk groups exhibited different genomic profiles, including aneuploidy score and purity score (Fig. 6, *E*–*H*).

## Experimental procedures

### Bulk transcriptome datasets

In this study, we curated OC bulk transcriptome datasets from GEO database, incorporating a total of 26 cohorts (18, 19, 20, 21, 22, 23, 24, 25, 26, 27, 28, 29, 30, 31, 32, 33, 34, 35, 36, 37, 38, 39, 40, 41, 42, 43). Detailed clinical information for these datasets is summarized in Table S1. The platforms utilized across these datasets included Affymetrix Human Genome U133A Array, Affymetrix Human Genome U133 Plus 2.0 Array, Agilent-014850 Whole Human Genome Microarray, and Operon Oligonucleotide Microarray. mRNA expression profiles were extracted and analyzed from these datasets. Among the curated datasets, 11 cohorts included information on overall survival, while three cohorts provided data on progression-free survival or disease-free survival. Additionally, for validation purposes, we acquired mRNA expression profiles and clinical data for OC patients from TCGA *via* the UCSC Xena project.

### Identification of consensus molecular subtypes

Based on 26 cohorts from GEO database, we implemented a computational framework to identify consensus molecular subtypes. Initially, we integrated expression data from common genes across these datasets, which were obtained from four distinct platforms, using the combat method (44). The resulting merged expression matrix was designated as the GEO merged dataset. Next, we identified the top 1500 genes with the highest variability across the 1987 samples in the merged dataset using the median absolute deviation strategy. Subsequently, we applied consensus non-negative matrix factorization clustering to reduce the dimensionality of the expression data from thousands of genes to a set of metagenes within subclass of the dataset (45). This approach involves computing multiple k-factor factorization of the expression matrix and assessing solution stability using the cophenetic coefficient. To enhance robustness, we computed silhouette widths to exclude samples that were not robust representatives of their respective subclasses. The remaining samples within each subclass were then classified into distinct molecular subtypes based on their expression profiles.

### Module analysis for each molecular subtype

Based on the merged dataset, we employed MEGENA method to delineate coexpressed modules specific to each molecular subtypes (46). MEGENA, an R package for multiscale embedded gene coexpression network analysis, offers enhanced performance compared to other coexpression analytical tools. The MEGENA framework encompasses the construction of a planar filtered network, multiscale clustering analysis, and subsequent downstream investigations.

For each molecular subtype, we selected genes with a gene weight greater than 5.5 and an edge weight exceeding 0.6 to construct subtype-specific network modules. Additionally, we utilized the limma package (47) to identify genes upregulated in each subtype relative to the other three subtypes (log fold change [FC] > 1, false discovery rate (FDR) < 0.05; TT), and relative to normal samples (logFC > 1, FDR < 0.05; TN). Subsequently, we assessed the overlap between MEGENA modules and TT/TN genes for each subtype using hypergeometric test, identifying specific TT and TN modules with a significance threshold of *p* value < 0.05. The overlap between TT and TN modules was also determined. In cases where multiple modules exhibited topology relationships, the module demonstrating the most significant association was designated as the final subtype-specific module.

### Inference of cancer- and stromal-specific expression

Tumor samples consist of both cancerous and stromal cells. We implemented a framework to estimate cancer-specific and stromal-specific expression profiles for each molecular subtype based on the GEO merged dataset. Initially, we assessed tumor purity using the "estimate" R package (15) for each sample in the merged dataset. Subsequently, we employed least squares regression to infer the expression levels of each gene in the cancer and stromal compartments within each subtype (48). The subtype mRNA expression $e_{\text{tumor},i}$, representing the combined expression across cancer and stromal cells for a given gene in sample i, can be formulated as follows:

$$e_{\text{subtype},i}=p_{i}\times \bar{e_{\text{cancer}}}+\left(1-p_{i}\right)\times \bar{e_{\text{stroma}}}$$

where p_i_ represents the proportion of cancer cells (tumor purity) in sample i. And $\bar{e_{\text{cancer}}}\;\text{and}\;\bar{e_{\text{stroma}}}$ denote the average expression of the gene in the cancer and stromal compartments, respectively. Using this formula, we derived cancer-specific and stromal-specific expression profiles for each gene within each molecular subtypes.

### LR interaction analysis

We obtained 1400 LR pairs from a prior paper (49). The product of ligand and receptor gene expression was used to estimate the relative flow of signaling between cancer and stromal compartments across four subtypes. The product of ligand and receptor gene expression was used to estimate LR complex activity (48). Four possible directions, cancer-to-cancer, cancer-to-stromal, stromal-to-cancer, and stromal-to-stromal were considered. Take the cancer-to-cancer (C>C) as an example, the RC score was calculated based on relative complex concentration given all four directions as follows within each molecular subtype:

$$S_{subtype,c>c}=\frac{e_{L,C}\times e_{R,C}}{e_{L,C}\times e_{R,C}+e_{L,C}\times e_{R,S}+e_{L,S}\times e_{R,C}+e_{L,S}\times e_{R,S}}$$

For other three directions, the similar formula was utilized for RC score calculation. Moreover, we derived the comprehensive RC score across all molecular subtypes as the average value of four subtype scores.

### Subtype-specific genes identification

To identify the most robust genes for each molecular subtype, we acquired four gene sets and defined the intersection of four genes sets as subtype specific genes. Gene sets 1 and 2 encompassed previously defined TT and TN genes, respectively, which were identified by limma package with logFC > 1 and FDR < 0.05 based on the merged bulk dataset. Utilizing estimated cancer-specific compartment profiles, we calculated the FC value between one subtype and other three subtypes, resulting in the selection of the top 200 genes for gene set 3. Additionally, we identified another set of top 200 genes exhibiting high FC values between this subtype and normal samples, designated as gene set 4. And the cut-off 200 for gene set 3 and 4 was defined based on the maximum gene number of gene set 1 and 2. Ultimately, subtype-specific genes were determined as the intersection of these four gene sets for each molecular subtype.

### Subtype score for malignant epithelial cells

We retrieved three OC single-cell transcriptome datasets from the GEO database (GSE118828, GSE130000, and GSE146026) (50, 51, 52). Raw data were processed to construct Seurat objects, and dimensionality reduction clustering was performed using the Seurat (4.2.0) R package (53). To identify cell types, we employed the SingleR (1.10.0) method (54) with reference to the HumanPrimaryCellAtlasData and utilized the infercnv (1.12.0) R package to identify malignant epithelial cells.

Based on the expression profiles of malignant epithelial cells, the expression levels were quantified as E_ij_ for gene i in cell j. Given a set of genes (G_subtype_) reflecting gene signature unique to each molecular subtype, we calculated a score SC_subtype_(j) quantifying the expression of G_subtype_ in cell j, as the average expression of the genes in G_subtype_, compared to the average expression of a control gene-set (G_control_) as follows:

SC(j) = average[E(G_subtype_,j)]-average[E(G_control_,j)]

For the control group G_control_, all analyzed genes were initially categorized into 30 bins. If the total number of genes analyzed did not evenly into 30 bins, any remaining genes were allocated to the 30th bin. Subsequently, we identified the bin containing G_subtype_ and randomly selected 100 genes from each of these bins to form the control gene set. This ensured that the expression distribution of control gene set closely mirrored that of G_subtype_, albeit scaled up by a factor of 100. In the preceding section, we computed scores for each malignant epithelial cell based on four subtype-specific signatures, facilitating the classification of cells into four distinct subtypes with the highest subtype score. And the cells were defined as subtype hybrids and removed that meet the following criteria: (i) the score of the second subtype is greater than 1; (iii) the difference between the scores of the second and third subtypes is greater than 0.3; and (iii) the score of the second subtype is higher than 10% of the total number of mappings.

### Cell communication analysis

To investigate cellular interactions at the single-cell level, we utilized the iTALK (55) method to explore communication relationships among different cell types within molecular subtypes. Additionally, differential upregulated genes specific to each were identified using the FindMarkers function in the Seruat package. Furthermore, we utilized iTALK to investigate LR interactions between T cells and malignant cells across different molecular subtypes.

### Prognostic model construction

Significant prognostic differences were observed among OC patients across various molecular subtypes, particularly highlighting the C1 subtype (poorest prognosis) and C2 subtype (best prognosis). Thus, leveraging gene signatures from these subtypes, we developed a predictive model for OC prognosis. The training dataset consisted of a merged GEO dataset comprising 485 high-quality samples, while the TCGA dataset served as the validation set. Cox regression with the LASSO method (56) was employed to construct models for calculating the overall survival score (OSS) as follows:

OSS=0.0166×MFAP4+0.1858×FBLN2+0.0227×IGF2−0.0034×NME5−0.1435×MGLL−0.0101×COLEC11−0.0415×AGR2−0.0453×TFF3−0.0199×PAEP−0.0275×DEFB1

## Data availability

The data that support the findings of this study are available from the corresponding author upon reasonable request.

## Conflict of interests

The authors declare that they have no conflicts interest with the contents of this article.

## Figures and Tables

**Figure S6:**