# Supplementary material for: Comprehensive analysis of consensus molecular subtypes for ovarian cancer from bulk to single-cell perspectives
Source: J Biol Chem. 2024 Aug 22;300(9):107710. doi: 10.1016/j.jbc.2024.107710 (PMC11418113; doi:10.1016/j.jbc.2024.107710)
Supplement: Supplementary File Legends [file mmc1.docx]

## Supplementary Figure Legends

**Figure S1. Molecular subtype identification.** (**A**) Cophenetic coefficient results when k equals from 2 to 6. (**B**) Connective matrix results when k equals to 4.

**Figure S2. Subtype specific gene analysis.** (**A**) Number and associations of TT and TN genes within four subtypes. (**B**) Associations between TT top50 genes of four subtypes and pre-defined TCGA subtypes. (**C**) Network display of C1-885 module of C4 subtype and (**D-E**) Survival results within merged GEO and TCGA datasets.

**Figure S3. Protein interaction network for each molecular subtype.** Gene network with gene weight > 5.5 and edge weight > 0.6 for each molecular subtype (**A-D**) C1 - C4. Some representative genes were shown in the network. (**E**) The gene overlap of these four subtype networks.

**Figure S4.** **Expression profiles of ligands and receptors across molecular subtypes.** (**A**) C1-C4 expression of ligands or receptors in cancer and stromal compartments. (**B**) Overall expression in cancer or stromal compartments.

**Figure S5. Single-cell analysis and recurrence validation of candidate signatures.** Percentage distribution of subtypes in three single-cell datasets (**A**) GSE118828 (**B**) GSE146026 SS2 platform (**C**) GSE146026 10X platform. (**D**) The recurrence validation of candidate gene signatures in GSE44104 dataset.

**Figure S6. Subtype gene signatures in merged GEO dataset.** The expression heatmap of four subtype signatures in merged GEO dataset. TCGA subtype information was labeled for samples.

## Supplementary Table Titles

**Table S1.** The detailed information of merged GEO dataset.

**Table S2.** Module genes within each molecular subtype.

**Table S3.** Subtype signature genes.

**Table S4.** The representative differentially expression genes for each molecular subtype at single cell level (GSE130000).
