# Supplementary material for: Comprehensive analysis of consensus molecular subtypes for ovarian cancer from bulk to single-cell perspectives
Source: J Biol Chem. 2024 Aug 22;300(9):107710. doi: 10.1016/j.jbc.2024.107710 (PMC11418113; doi:10.1016/j.jbc.2024.107710)
Supplement: Table S1 [file mmc2.docx]

**Table S1. The detailed information of merged GEO dataset.**

| **Dataset** | **Platform** | **Sample Number** | | | **OS** | **Stage** | **PFS/DFS** |
| --- | --- | --- | --- | --- | --- | --- | --- |
|  |  | **Cancer** | **Normal** | **Sum** |  |  |  |
| GSE65986 | GPL570 | 55 |  | 55 | √ | √ |  |
| GSE63885 | GPL570 | 101 |  | 101 | √ | √ |  |
| GSE6008 | GPL96 | 99 | 4 | 103 |  | √ |  |
| GSE14764 | GPL96 | 80 |  | 80 | √ | √ |  |
| GSE29450 | GPL570 | 10 | 10 | 20 |  |  |  |
| GSE22600 | GPL570 | 15 |  | 15 |  |  |  |
| GSE14407 | GPL570 | 12 | 12 | 24 |  |  |  |
| GSE9891 | GPL570 | 285 |  | 285 |  | √ |  |
| GSE26193 | GPL570 | 107 |  | 107 | √ | √ | √ |
| GSE26712 | GPL96 | 185 | 10 | 195 | √ | √ |  |
| GSE32062 | GPL570 | 10 |  | 10 |  |  |  |
| GSE36668 | GPL570 | 8 | 4 | 12 |  |  |  |
| GSE18520 | GPL570 | 53 | 10 | 63 | √ | √ |  |
| GSE23391 | GPL570 | 3 | 5 | 8 |  |  |  |
| GSE52037 | GPL570 | 10 | 10 | 20 |  | √ |  |
| GSE27651 | GPL570 | 43 | 6 | 49 |  |  |  |
| GSE14001 | GPL570 | 20 | 3 | 23 |  |  |  |
| GSE15578 | GPL570 | 11 | 6 | 17 |  |  |  |
| GSE44104 | GPL570 | 60 |  | 60 |  | √ |  |
| GSE51373 | GPL570 | 28 |  | 28 |  | √ |  |
| GSE23554 | GPL96 | 28 |  | 28 | √ | √ |  |
| GSE3149 | GPL96 | 153 |  | 153 |  |  |  |
| GSE19829 | GPL570 | 28 |  | 28 | √ | √ | √ |
| GSE30161 | GPL570 | 58 |  | 58 | √ | √ |  |
| GSE17260 | GPL6480 | 110 |  | 110 | √ | √ | √ |
| GSE13876 | GPL7759 | 415 |  | 415 | √ | √ |  |
