# Supplementary material for: Comprehensive analysis of consensus molecular subtypes for ovarian cancer from bulk to single-cell perspectives
Source: J Biol Chem. 2024 Aug 22;300(9):107710. doi: 10.1016/j.jbc.2024.107710 (PMC11418113; doi:10.1016/j.jbc.2024.107710)
Supplement: Table S2 [file mmc3.docx]

**Table S2. Module genes within each molecular subtype.**

| **subtype** | **module** | **gene** |
| --- | --- | --- |
| C1 | C1_c1_303 | CD99;LGALS1;CRYAB;CD248;SNAI2;DYNLT3;PMM1;AVEN;PRKCDBP;HSPB2;OAT;TSPO;SEPW1;FBLN2;HEBP2;TMSB10 |
| C1 | C1_c1_345 | ACTG2;LMOD1;MYH11;CNN1;AOC3;TAGLN;MYLK;FLNA;KCNMB1;MYL9;FILIP1L;CSRP1;CES1;FRY;KIF13B;LPP;LIMS2;COX7A1;DES;HEPH;ALDH1B1;AMIGO2;CRYL1;SORBS1;KLF7;PPP1R12B;OSR2;RBPMS;FNBP1;ITGA8;SRF;FRYL;HK1;RAB11FIP1;PLN;TNS1;WFDC1;FLNC;TMEM47;SVIL;SLC22A3;MKNK1;PHYHIP;PDZRN4 |
| C1 | C1_c1_348 | COL6A1;COL6A2;MMP2;DACT1;PCOLCE;ARHGDIA;ADAM15;EMILIN1;CDC42EP1;RHOBTB2;RAI14;GLI3;HPCAL1;MAPKAPK2;SERPINH1;WBP2;SORBS3;NOS3;ST6GALNAC4;SDC3 |
| C1 | C1_c1_728 | ITGA5;GFPT2;PLAUR;MMP14;ADAM19;SLC11A1;ICAM1;ADAM8;IL1RN;CALB2;FOSL2;ANPEP;CA12;DNAJC15;MSC;TMEM2;SHB;RTN2;MSX2;IL1R2;TREM1 |
| C1 | C1_c1_766 | EGR1;DUSP1;FOS;CYR61;IER2;CTGF;CEBPD;GADD45B;EMP1;IER3;KLF6;EDN1;TIPARP;GADD45A;C8orf4;ADAMTS1;BCL6;LIF;VIM;ZFP36L1 |
| C1 | C1_c1_799 | SLC2A14;CFH;CTSK;FBN1;LXN;PALLD;DCN;NBL1;ECM2;MMP19;EDNRA;OLFML3;NT5E;LTBP2;ITGBL1;ASPN;FZD1;C1QTNF1;RGS4;DPYSL3;SEMA3C;CFHR1;ADRA2A;SLC2A3;NDN;LMCD1;GNGT1;LRRC17;SERPINF1;THBS1;SFRP4;OMD;PRRX1;SRPX2;TCF4 |
| C1 | C1_c1_801 | AEBP1;ACTN1;MYH9;CILP;MRC2;ISLR;KIAA0247;HIF1A;ACSL4;SPSB1;LBH;NCOR2 |
| C1 | C1_c1_1012 | COL1A1;COL1A2;COL3A1;COL5A1;COL5A2;POSTN;ADAM12;COPZ2;COL5A3;LOX;SERPINE1;LEPRE1;KDELR3;MXRA5;LOXL2;COL8A1;ANTXR1;GREM1;GJA1;RAB31;P4HA2;OLFML2B;ATP6V0E1;RUNX1;MAFB;FKBP14;GALNT1;EGFL6;PHLDA1;CHN1;EPYC;ARFGAP3;PLXDC2;PLXDC1;PLOD2;ASTN1;PXDN;OLFML2A;NXN;RIN2;KDELC1;SEMA5A;ITGB1;SLC20A1;FOXF2;DAP;RCN3;FN1;TGFBI;TDO2;THY1;KCNE4;PPIC;MAPK9;SCG2;UNC5B;SLC39A14;XYLT1;TPM4;VSNL1;SAMD4A;PLXNA2 |
| C1 | C1_c1_1015 | COL11A1;COL10A1;DOK5;COL8A2;MATN3;COMP;MOXD1;CYP26A1;CMTM6;BBOX1;ITGB5;SPP1;ST6GALNAC5 |
| C1 | C1_c1_1019 | PLAU;ECM1;SLC12A8;PDPN;CDCP1;EGLN3;NOX4;PML;KAL1;ERO1L;VDR;TMEM158;UNC93B1;TNFRSF12A;PPFIBP1 |
| C2 | C2_c1_200 | SCGB1D2;ASRGL1;MPPED2;EYA2;EHF;KIAA1324;ESR1;SCGB2A1;HOMER2;SCGB2A2;SCGB1D1;AMPH;SOX17;FAM111A;SOX5;SLC15A2 |
| C2 | C2_c1_208 | DUSP4;MGLL;LMNA;ITGB3;FOXC1;NPTX2;GJB1;NPAS2;CAMK2N1;GCHFR;MMD;ABCC3;S100A6;LRP5L;SLC14A1;PDE9A;PRKCH |
| C2 | C2_c1_209 | DUSP6;ETV4;ETV5;SPRY1;SPRY2;KCNN4;ETV1;CCND1;SPRED2;INPP5F;HMGA2;SOX9;SPRY4;TRIB2 |
| C2 | C2_c1_210 | CD55;B3GNT3;CLDN10;CARD10;MMP10;NCALD;LDLR;PCTP;PAEP;SPINT1;UPK1B |
| C2 | C2_c1_219 | CXCL2;CXCL1;BIRC3;CCL20;CXCL3;NFKBIE;PDZK1IP1;ELF3;RELB;CXCL6;MMP7 |
| C2 | C2_c1_356 | NDUFS2;IARS2;KIFAP3;PPOX;CYB5R1;KRT23;GYG2;TFB2M;UFC1;PPAP2C |
| C2 | C2_c1_548 | ALPP;CHGA;MUC5B;A4GALT;CR1;IL20RA;ALPPL2;KCNQ1;RGS17;PTPRU;NRTN |
| C2 | C2_c1_871 | GALNT7;ENTPD3;ARFIP2;TFF3;RAB6B;AQP5;DUSP9;TRIM2;SERPINA1;PPAP2B |
| C3 | C3_c1_397 | AANAT;GBX2;CACNA1F;CDX4;CRX;OPN1LW;NMUR1;FSHR;IL4;OPN1SW;NPBWR2;HCRTR1;ARSF;DRD1;KRT36;CRYGA;KCNH1;RCVRN;SOX14;AVP;KCNA3;APOF;COL19A1;HRC;PDE6H;OR1A2;GABRA6;WNT1;GPR50;GPR31;FETUB;CRYBA1;IFNA8;CYP2F1;MYH1;GYS2;MBL2;TPO;TAS2R7;RXFP3;ZBTB32;SLC2A4;ZNF157;PRDM9;HTR1A;SCT;POU4F3 |
| C3 | C3_c1_622 | LAPTM5;C1QA;APOE;IFI30;AXL;MAFB;CTSC;GPNMB;NAGK;CAPG;HMOX1;GLUL;GRB2;NAGA;STIM1;PSAP;PIK3R2;TPP1 |
| C3 | C3_c1_625 | IL2RB;GZMB;NKG7;GNLY;CD7;KLRD1;CTSW;LAG3;KLRC1;IL15;CCDC109B;INPP4B;PRF1;SOCS1 |
| C3 | C3_c1_633 | POU2AF1;PIM2;IGJ;LAX1;IRF4;CKAP2;EAF2;FAM46C;CD38;FKBP11;AMPD1;SLAMF7;TNFRSF17;FBXW7;STARD5;ZNF215;RAB33A |
| C3 | C3_c1_955 | ISG15;MX1;IFI6;OASL;IRF7;IFI27;BST2;IFI44;ZC3HAV1;PLEKHA4 |
| C3 | C3_c1_958 | CD2;CCL5;GZMA;KLRK1;CD247;CXCL9;APOBEC3G;PSTPIP2;TARP;ITM2A;RASGRP1;PARP8 |
| C3 | C3_c1_959 | CD3D;CD52;CD8A;GZMH;CST7;BATF;ICAM2;CXCL13;VAMP5;ITM2C |
| C3 | C3_c1_1000 | DUSP1;CYR61;ATF3;RGS1;RGS2;CD69;KLF6;DUSP2;DUSP5;BTG2;PHLDA1;C8orf4;IER3;ZFP36;SERPINE1;SOCS3;GADD45A;S100A10;STK17B |
| C4 | C4_c1_534 | RNPS1;MRPL28;KCTD5;CREBBP;C16orf59;HCFC1R1;GPRC5B;ITM2C;CLDN6;PIGQ;THOC6;NME4;CCNF;IGF2;UBE2I;ZNF263;IQCK;SERPINH1 |
| C4 | C4_c1_716 | AP2M1;POLR2H;ABCF3;PARL;MAGEF1;EIF2B5;FXR1;PSMD2;DVL3;EIF4G1;ALG3;MCCC1;HMGA2;KLHL24;IGF2BP2;DENND1B;ABCC5;CDK9;CXCL12;CLCN2;SKIL;USP13;RPL39L;YEATS2;VPS8;SEC61A1;FLNA;TSC22D3;TBL1X |
| C4 | C4_c1_885 | FSCN1;MARCKSL1;LRP4;DACH1;C11orf49;IL12A;FYN;PLCB4;PALLD;TMEM45A;TSC22D1;PHF21A;SRGAP2;SOX9 |
| C4 | C4_c1_973 | BMP7;FGF18;NEDD9;MFHAS1;BCAT1;GDF11;LHX1;WNT7A;PDE9A;NR3C1;LMO3;SIX3 |
| C4 | C4_c1_1089 | CDCA3;NCAPD2;EMG1;NDUFA9;DERA;C12orf4;DYRK4;ITFG2;RAD51AP1;DDX11;PLK1;TMPO;PLEKHA5;ACBD4;NINJ2 |
