# Supplementary material for: Comprehensive analysis of consensus molecular subtypes for ovarian cancer from bulk to single-cell perspectives
Source: J Biol Chem. 2024 Aug 22;300(9):107710. doi: 10.1016/j.jbc.2024.107710 (PMC11418113; doi:10.1016/j.jbc.2024.107710)
Supplement: Table S3 [file mmc4.docx]

**Table S3. Subtype signature genes.**

| **C1** | **C2** | **C3** | **C4** |
| --- | --- | --- | --- |
| MFAP4 | MUC5B | CXCL10 | PLAC1 |
| COL5A1 | NME5 | LAMP3 | CACNA2D2 |
| NUAK1 | MGLL | IFI27 | HMGA2 |
| COL1A1 | COLEC11 | ISG15 | MEST |
| COL8A2 | DUSP4 | VTCN1 | NEK2 |
| FBLN2 | AGR2 | CEP55 | RAD51AP1 |
| MFAP2 | NPDC1 | HTR3A | BMP7 |
| PXDN | PPAP2C | CDKN2A | LHX1 |
| TCF7L1 | KCNN4 | IFI6 | LRP4 |
| COMP | TFF3 | KLK7 | CLDN6 |
| CXCL14 | KRT23 | BBOX1 | LGR5 |
| MMP11 | TSPAN1 | CP | SOX11 |
| MFAP5 | MLPH | LCN2 | ZIC1 |
| IGF2 | C6 | DEFB1 |  |
| SOX11 | GCNT3 | CACNA1F |  |
|  | FCGBP | WNT1 |  |
|  | SCGB2A2 | HCRTR1 |  |
|  | PAEP |  |  |
|  | LCN2 |  |  |
|  | CLDN10 |  |  |
|  | DEFB1 |  |  |
|  | RAB25 |  |  |
|  | SERPINA5 |  |  |
|  | SCGB2A1 |  |  |
