# Supplementary material for: Comprehensive analysis of consensus molecular subtypes for ovarian cancer from bulk to single-cell perspectives
Source: J Biol Chem. 2024 Aug 22;300(9):107710. doi: 10.1016/j.jbc.2024.107710 (PMC11418113; doi:10.1016/j.jbc.2024.107710)
Supplement: Table S4 [file mmc5.docx]

**Table S4.** The representative differentially expression genes for each molecular subtype at single cell level (GSE130000).

| **Subtype 1** | | **Subtype 2** | | **Subtype 3** | | **Subtype 4** | |
| --- | --- | --- | --- | --- | --- | --- | --- |
| **Up** | **Down** | **Up** | **Down** | **Up** | **Down** | **Up** | **Down** |
| CXCL14 | CDKN2A | SCGB2A1 | CXCL8 | IFI27 | LGR5 | LGR5 | CDKN2A |
| COL1A1 | TSPAN1 | TSPAN1 | CRABP2 | CP | S100A2 | MEST | TSPAN1 |
| MMP11 | CLDN10 | AGR2 | ISG15 | KLK7 | TSPAN1 | RAD51AP1 | CLDN10 |
| MFAP5 | CP | AGR3 | NTS | IFI6 | CCDC146 |  | ISG15 |
| PXDN | ISG15 | MGLL | CDKN2A | ISG15 | MGST1 |  | KLK7 |
|  | IFI6 | MGST1 | SST | CDKN2A | KCNQ1OT1 |  | IFI6 |
|  | KLK7 | MS4A8 | IFI6 | NTS | MS4A8 |  | CP |
|  | SCGB2A1 | CCDC146 | CP | SST | FHAD1 |  | SCGB2A1 |
|  | IFI27 | SPAG17 | KLK7 |  | CAPS |  | IFI27 |
|  |  | UBB | IFI27 |  | AGR3 |  |  |
|  |  | SCGB1D4 |  |  | CFAP43 |  |  |
|  |  | TPPP3 |  |  | SPAG17 |  |  |
|  |  | C11orf88 |  |  | UBB |  |  |
|  |  | S100A4 |  |  | SCGB2A1 |  |  |
|  |  | S100A2 |  |  |  |  |  |
|  |  | FHAD1 |  |  |  |  |  |
|  |  | RSPH1 |  |  |  |  |  |
|  |  | NAPSB |  |  |  |  |  |
|  |  | RASD1 |  |  |  |  |  |
|  |  | PIFO |  |  |  |  |  |
|  |  | LRRIQ1 |  |  |  |  |  |
|  |  | NPDC1 |  |  |  |  |  |
